# Maternal Health Is Women's Health: A Call for Papers for Year 2 of the Maternal Health Task Force–PLOS Collection

**DOI:** 10.1371/journal.pmed.1001350

**Published:** 2012-11-27

**Authors:** Samantha R. Lattof, Mary Nell Wegner, Ana Langer, the PLOS Medicine Editors

**Affiliations:** 1 Harvard School of Public Health, Global Health and Population, Boston, Massachusetts, United States of America; 2 Harvard School of Public Health, Women and Health Initiative, Boston, Massachusetts, United States of America

## Abstract

The *PLOS Medicine* editors and the Maternal Health Task Force announce a new call for papers on the theme of “maternal health is women's health.”

The Maternal Health Task Force (MHTF) and the *PLOS Medicine* Editors are delighted to announce our continued collaboration to create a freely available, open access collection of outstanding research and commentary on maternal health, the MHTF–PLOS Collection on Maternal Health, with a call for papers on the theme of “maternal health is women's health.”

This Year 2 Collection follows the successful first year of our partnership, which generated a collection of 18 original articles on the quality of maternal health care (www.ploscollections.org/maternalhealth) and helps us further our collective goal to improve women's and children's health worldwide through greater access to more comprehensive maternal health information and knowledge. Our continued commitment to highlighting and pressing for more evidence on maternal health reflects the persistence of this problem around the world: while the number of maternal deaths overall is declining, Millennium Development Goal 5 (to improve maternal health) is the goal most lagging behind due to a lack of progress in a critical number of countries [1],[2].

We chose the theme for Year 2 of “maternal health is women's health” because it is crucial to consider maternal health in the context of women's health throughout their lifespans. While pregnancy is limited to women of reproductive age, maternal health is influenced by the health of women and girls before pregnancy, and it also influences women's health broadly during and after the reproductive years. Inability to access quality health care and family planning resources, low educational attainment, low socioeconomic status, restrictive gender roles, poor nutrition, and a host of other social and biological factors combine to put girls and women at risk for not being able to attain and sustain the health status they deserve throughout their lives.

## Key Issues in the Health of Girls and Adolescents

Even prior to puberty, a number of childhood factors exist that may influence girls' future pregnancies. Poor nutrition, vitamin and mineral deficiencies, illiteracy, the low social status of women, poverty, and cultural practices such as female circumcision, rationing food to female children, and child marriage threaten girls' health and increase in pregnancy the risk of obstructed labor, fistula, obstetric emergencies, and HIV infections, among other complications [3]–[5]. Younger women are at greater risk than other women of pregnancy complications because their bodies are still maturing: inadequately developed pelvises and short stature, influenced by childhood nutrition and poverty, often complicate early childbirth [6].

Girls are often not free to refuse sex or negotiate sex and condom usage, which increases their risk of sexually transmitted infections (STIs), including HIV. For example, the likelihood of being infected with HIV among women aged 15–24 years and living in sub-Saharan Africa is eight times that of men [7]. All but 5% of adolescent births occur in developing countries, where complications related to pregnancy and childbirth are a leading cause of death for girls aged 15–19 years [8]. For women of all ages, HIV/AIDS is an increasingly important indirect cause of maternal death, in part because of the increasing number of infections among sexually active women and because the proportion of women living with HIV/AIDS is increasing [7].

## Key Issues in the Health of Women

Once a woman becomes pregnant, she is at increased risk of HIV because of both her physical susceptibility and her relative disempowerment [9]. Two in five women in Lesotho, for example, report that they have no right to refuse sex with their partner, and almost half of men in Lesotho agree [10]. Gender-based violence is a key determinant of conditions such as HIV infection, pregnancy, and traumatic gynecologic fistula. Fistula can result in lasting incontinence, infections, ulcerations, infertility, and social stigma so severe it results in a woman being essentially exiled.

The links between unintended pregnancy and HIV affirm the importance of access to family planning. Meeting the unmet need for family planning, which includes access to contraception and safe abortion, would reduce the number of unintended pregnancies from 75 million per year to 22 million per year and assist in the reduction of maternal morbidity and mortality [11]. Importantly, commitments to provide family planning recognize women's sexual and reproductive rights, which are vital to women's health and dignity and thus essential to genuine progress in maternal health [12].

Non-communicable diseases brought on by pregnancy, such as pre-eclampsia/eclampsia and gestational diabetes, may leave women managing chronic conditions for the rest of their lives. Gestational diabetes, when unmanaged, places women at increased risk of developing diabetes mellitus (type 2) and places future pregnancies at risk of pre-eclampsia and emergency cesarean section [13]. Long-term and untreated complications of pregnancy and delivery, such as prolapse and fistula, affect the quality of life of hundreds of thousands of women around the world. Maternal mental health is also a growing global concern, with new estimates suggesting mental disorders may affect up to a third of mothers in some settings [14]. Untreated maternal mental illness affects infant and child growth and the quality of child care, resulting in compromised child development [15],[16].

## Call for Papers

For Year 2 of the MHTF–PLOS Collection on Maternal Health, we welcome primary research articles (both quantitative and qualitative) and incisive commentary related to maternal health in the context of women's health throughout their lifespan, and more specifically in the following areas that have been identified by the global maternal health community as critical needs (see Box 1):

1. maternal health as part of sexual and reproductive health issues (e.g. family planning, gender-based violence, STIs),
2. maternal health and non-communicable diseases (e.g. cardiovascular disease, mental health),
3. maternal health and communicable diseases (e.g. malaria, HIV/AIDS),
4. implications of child and adolescent health for maternal health, and
5. consequences of poor maternal health in later stages of women's lives (e.g. prolapse and other chronic morbidities).

**Box 1: Year 2 MHTF–PLOS Collection on Maternal Health: Call for Papers**

We welcome primary research and incisive commentary related to maternal health in the context of women's health throughout their lifespans. Specific priorities are:

1. Maternal health as part of sexual and reproductive health issues (e.g., family planning, gender-based violence, STIs)
2. Maternal health and non-communicable diseases (e.g., cardiovascular disease, mental health)
3. Maternal health and communicable diseases (e.g., malaria, HIV/AIDS)
4. Implications of child and adolescent health for maternal health
5. Consequences of poor maternal health in later stages of women's lives (e.g., prolapse and other chronic morbidities)

Research articles should not be merely descriptions of activities but should include evaluation of the impact of initiatives after their implementation. Commentary articles on maternal health in the context of women's health throughout the lifespan, targeted to the *PLOS Medicine* Essay, Policy Forum, or Health in Action sections, must be novel and well-argued. Authors should refer to the *PLOS Medicine* Guidelines for Authors at http://www.plosmedicine.org/static/guidelines.action for specific submission requirements.

All papers should be submitted to *PLOS Medicine*, with a note that they are intended for the Maternal Health Collection. Authors for whom the publishing fee for research articles presents a barrier are encouraged to select the PLOS fee waiver when submitting an article. An initial decision will be made about papers' potential suitability for either *PLOS Medicine* or another PLOS journal. The authors will be informed of this decision, and papers considered appropriate for PLOS journals will then be peer-reviewed according to the specific journal's policies; no articles can be guaranteed of acceptance at any journal. PLOS editors will retain all control over editorial decisions. Editorial staff has no knowledge of an author's ability to pay publication fees, so ability to pay cannot affect decisions. If and when a paper is accepted for publication in a PLOS journal it will be forwarded to the selection panel for the Collection. This panel, which is composed of PLOS and MHTF staff, will decide on articles' suitability for inclusion in the Collection.

We invite submissions now on the theme “maternal health is women's health”—maternal health in the context of women's health throughout their lifespan. Articles will stand the best chance of inclusion in the Year 2 Collection if they are submitted by 01 April 2013.

PLOS and the MHTF look forward to our continued collaboration on this initiative. We hope it will encourage researchers to submit to *PLOS Medicine* their best maternal health research and commentary.

## Abbreviations

MHTF: Maternal Health Task Force
STI: sexually transmitted infection
